# Impact of length and width of footwear on foot structure of preschool-aged children

**DOI:** 10.7717/peerj.13403

**Published:** 2022-05-03

**Authors:** Ewa Puszczalowska-Lizis, Sabina Lizis, Magdalena Prusak, Jaroslaw Omorczyk

**Affiliations:** 1 Medical College, Institute of Health Sciences, University of Rzeszow, Rzeszow, Poland; 2 Health Center “Tuchow”, Tuchow, Poland; 3 Faculty of Physical Education and Sport, Institute of Sport, University of Physical Education in Krakow, Krakow, Poland

**Keywords:** Indoor shoes, Footwear fit, Foot, Girls, Boys

## Abstract

**Background:**

Due to the dynamics of developmental changes, the preschool age is of crucial importance for the later health and efficiency of the feet. The aim of this study was the analysis of the fitting of indoor footwear and its impact on the features of the foot structure in 6-year-old children.

**Methods:**

The study group consisted of 100 children, including 50 girls and 50 boys at the age of 6 years. The applied device was the CQ-ST podoscope and the Clevermess. The data were analyzed based on Mann-Whitney *U* test, Chi-square test and regression analysis.

**Results:**

About 60% of children wore correctly fitted shoes in terms of length and width. Multiple regression models with two variables explaining the variance of the Clarke’s angle were statistically significant for girls (right foot: *p* < 0.001 and left foot: *p* = 0.009), and boys (right foot: *p* < 0.001 and left foot: *p* < 0.001). The influence of predictive variables on the values of the heel angle (*γ*) was statistically significant for girls (right foot: *p* < 0.001 and left foot: *p* < 0.001) and boys (right foot *p* < 0.001 and left foot: *p* < 0.001).

**Conclusions:**

Both in the case of girls and boys, the frequency of using too long and too wide shoes was higher in relation to the frequency of using too short and too narrow shoes. The length and width of the shoes affected the length and width of the footwear both in girls and boys. The longer and wider the shoes, the lower the height of the arch. Longer shoes are accompanied by a greater transverse arch, and wider ones with a lower transverse arch of the foot.

## Introduction

The human foot is an important supporting and weigh bearing component of the musculoskeletal system which has its unique structure. During ontogenetic development, it undergoes numerous changes that prepare it for weight bearing and locomotion. The most important period from the perspective of foot formation is the pre-school period, characterized by high intensity of developmental changes (Matsuda et al., 2012; Prętkiewicz-Abacjew & Opanowska, 2013; Yurt, Sener & Yakut, 2014; Vrdoljak & Tiljak, 2017). At the age of 3, the process of arch shaping is initiated, and the longitudinal and transverse arches become clearly visible at the age of 6 (Jiménez-Ormeño et al., 2011; Müller et al., 2012; Jiménez-Ormeño et al., 2013; Barisch-Fritz, Plank & Grau, 2016; Carr, Yang & Lather, 2016; Medina-Alcántara et al., 2019). At that age, muscles, tendons and ligaments as well as connective tissue in children’s foot increase in strength also developing foot stability. The foot muscles still lack adequate strength, therefore the leg swing force is slight with the ankle becoming weak and unstable, which may result in foot disease and deformity. The exaggerated pressure on the arch may result in flat feet, thus this stage is referred to as the fragile growth period of the foot (Zhang & Wang, 2017).

Due to the dynamics of developmental changes, the preschool age is of crucial importance for the later health and efficiency of the feet. Therefore, the child’s foot during this period requires special attention. Delgado-Abellán et al. (2014) stressed that the development of the foot is influenced by both biological factors, such as age, sex, as well as extra-biological factors, which include the type of footwear worn. Klein et al. (2009) emphasized that during this period children spend most of the day in kindergarten, wearing indoor footwear. Therefore, monitoring shoes fitting to their feet is of particular importance. Such a procedure will allow for frequent replacement of shoes, and thus will reduce the incidence of foot deformities.

In terms of footwear fitting, an important issue is the so-called functional excess. In the case of the shoe length, it is a free space between the tip of the shoe and the longest toe in the footwear (Knapik, 2000; Müller et al., 2012; Herbaut et al., 2019; Kinz, Groll-Knapp & Kundi, 2021). On the one hand, it provides a reserve for growing feet, and on the other, it secures space for the so-called apparent increase in the length of the foot, resulting from the elongation of its longitudinal arch during locomotion or load. The functional excess should take into account the average foot increase rate in the period from 6 to 12 months (Vrdoljak & Tiljak, 2017). The comfort of footwear use also depends to a large extent on its fitting in relation to the foot width. The functional excess of the width of the footwear is a space for the so-called apparent widening of the forefoot due to free strain of its transverse arch (Rajchel-Chyla et al., 2012; González Elena & Córdoba-Fernández, 2019). According to Chaiwanichsiri, Tantisiriwat & Janchai (2008) wearing shoes that are too narrow, especially in childhood, causes abnormal tension in the muscles and tendons of the feet, deformation of the toes, corns and unsightly callouses. Too narrow shoes put pressure on the feet, making it difficult for the muscles to work, inhibiting natural development. In turn, the use of shoes that are too wide may be the reason of instability during locomotion and resulting injuries (Penkala et al., 2011; Pavlackova et al., 2015; Lim et al., 2015; Dinato et al., 2015; Hettigama, Punchihewa & Heenkenda, 2016; Hellstrand Tang et al., 2017; Herbaut et al., 2017; González-Elena et al., 2021).

The analysis of the extensive literature indicates that shoes fitting in relation to the developing foot is an area with a limited scope of empirical study. This was the reason for undertaking the subject of the study, the aim of which was reduced to the analysis of the fitting of indoor footwear and its impact on the features of the foot structure in 6-year-old children. In order to thoroughly analyze this issue, we undertook to investigate whether gender differentiates the values of foot structure indices and if it is a factor determining the frequency of longitudinal and transverse arch deformation, as well as the position of the hallux and fifth toe in preschool-aged children. Another issue was to prove whether there are intergender differences in terms of the functional length excess and the functional width excess of the footwear worn by the studied children, as well as what is the frequency of wearing footwear adequately fitted for length and width and is it determined by gender. An important issue was to investigate whether the size of the functional length and width excess in indoor footwear affect the features of the feet structure of preschool-aged children.

## Material & Methods

### Participants

The cross-sectional study included 100 children aged 6 attending randomly selected kindergartens in the commune of Tuchow in the south-eastern Poland.

The inclusion criteria were the age of 6, regular attendance at the kindergarten, staying in the preschool institution for at least 7 h a day, wearing indoor footwear on the premises of the kindergarten, understanding the instructions that were necessary for the measurement procedures, and written informed consent of parents or guardians to participate in the study.

The exclusion criteria included children from preterm deliveries and those with lower limb deformities, neurological diseases and after diseases and/or injuries of the musculoskeletal system, including lower limbs. The basis for exclusion from the study was also the refusal or unwillingness of the child to cooperate during the implementation of research procedures.

Calculations taking into account the 95% confidence level and the 5% acceptable fraction estimation error indicated that the sample size should include 122 subjects. After the allocation procedure, it was found that 22 children were excluded from the study protocol due to their non-compliance with the inclusion criteria. The remaining individuals were divided into two equal-sized groups including 50-person on the grounds of the gender.

### Protocol

The research tool was the podoscope CQ-ST (Electronic System, Ltd., EU). The device was recorded under PL/DR 009932 reference number in the *Register of Medical Devices and Corporate Entities in Charge of their Marketing and Use for Medical Purposes.* The procedure of stringent calibration during the manufacturing process is a guarantee of consistent accuracy of the readouts. The correction of geometric distortions ensures the accuracy of image reproduction up to one mm. The device was awarded a Declaration of Conformity (No. DS.05.2014), which guarantees its full compliance with the requirements stipulated in the Ordinance of the Minister of Public Health on the medical devices (Journal of Laws of 2003, No. 4, Item 45), issued on the basis of the provisions of Article 12 (2) and Article 16 (3) of the Act of July 27, 2001, on medical devices (Journal of Laws No. 126, Item 1380, and of 2002, No. 152, Item 1264).

The study included the measurement of the plantar feet surfaces in standing, with even distribution of body weight on both feet. The width and foot angle were natural, unforced. The calculations included the following indices: foot length, foot width, Clarke’s angle (longitudinal foot arch), heel angle *γ* (transverse foot arch), hallux valgus angle *α*, the angle of the varus deformity of the fifth toe *β* (Chen et al., 2011; Pita-Fernández et al., 2015; Puszczalowska-Lizis et al., 2021).

The fit of the footwear was tested with the Clevermess device (Clevermess Ltd., Titting, Germany). The measuring tool reliability is excellent: ICC = 0.993 for measurements of the functional excess of the footwear length and ICC = 0.992 for measurements of the functional excess of the footwear width (Puszczalowska-Lizis et al., 2020). Footwear was found to be well-fitted in length when the value of the functional excess for length was in the range from 8 to 12 mm, while the appropriate fit in width was found when the functional excess for width was in the range from 1 to 3 mm (Knapik, 2000; Puszczalowska-Lizis et al., 2020).

The assessment took into account gender-specific footwear of a certain brand (Befado). They were the participants’ own shoes, purchased by their parents. Children wore the same model of footwear, but the shoes dedicated to girls had white soles (catalogue no. 273X340), while boys’ shoes—navy blue (catalogue no. 273X321). Parents were recommended to choose this model of footwear, due to the relatively low price and high quality, especially in the context of functional and health features. These are light, linen slippers with a stiff heel, a widened toe, on a flexible, non-slip sole, fastened with Velcro. The tested footwear was used in girls from 1.00 to 4.00 months (2.04 ± 0.76 months), while in boys from 1.00 to 3.50 months (2.02 ± 0.74 months).

The tests were carried out in preschool institutions, in rooms intended for exercise and motor games, in the presence of children’s educators. The facilities included were full-time. The mean time spent by the children in the facilities was from 7:00am to 4:30pm on weekdays, with individual based variations. To maintain the reliability of the study process, measurements were taken in the morning, from 8.00 to 10.00 AM. Parents were informed about the date of the examination and were advised to ensure that the children did not undertake vigorous physical activity during 12 h preceding the examination. Before the study, the parents or legal guardians signed a declaration that they had complied with the above recommendation. Measurements were taken with the same instruments operated by the authors of the study. During the study the children were in their underwear. The podoscopic examination required removing socks and shoes, while children wore socks during the measurement of shoe fitting. The study was conducted in accordance with the Declaration of Helsinki. The research was carried out following endorsement by the Bioethics Review Committee, University of Rzeszow (Resolution no. 3/12/2015), and after obtaining written consent from the children’s parents or legal guardians. All respondents and their parents or legal guardians were advised of the actual purpose and key principles of the study, as well as on their statutory right to opt out of the study protocol at any stage.

### Analysis

The normal distribution of the values was verified with the Shapiro–Wilk test. In order to evaluate intergender differences in foot structure indices of the studied girls and boys we used the Mann–Whitney *U* test. Qualitative data analysis was performed using the Pearson Chi-square test. The influence of independent variables (predictive, explained, such as: functional length excess and functional width excess) on the dependent variables (criterial, such as: foot structure indices) were estimated on the basis of regression analysis after checking whether the residual distributions (differences between the observed value and calculated from regression equations) are normally distributed. The results were considered statistically significant, if the probability level of the test was lower than the predetermined significance level *p* < 0.05. The STATISTICA StatSoft, Inc., version 13.1 was used to process the test results.

## Results

Data in Table 1 show statistically significant inter-gender differences in the values of the Clarke’s angle (right foot: *p* = 0.001; left foot: *p* < 0.001). Girls had higher values of Clarke’s angle than boys.

**Table 1:** Comparison of the foot structure indices of the studied children.

Foot	Gender	$\bar {x}$± SD	Max–min	Q_25_	Me	Q_75_	Mann–Whitney *U* test
Foot length [cm]							
Right	Girls	17.59 ± 0.84	19.30–15.80	17.00	17.50	18.00	*Z* = − 0.64*p* = 0.519
	Boys	17.71 ± 0.91	19.80–15.90	17.00	17.70	18.50	
Left	Girls	17.55 ± 0.82	19.30–15.80	17.00	17.50	18.00	*Z* = − 0.95*p* = 0.341
	Boys	17.71 ± 0.91	19.80–15.90	17.00	17.70	18.50	
Foot width [cm]							
Right	Girls	6.82 ± 0.46	8.00–6.00	6.50	6.80	7.10	*Z* = − 1.59*p* = 0.109
	Boys	6.96 ± 0.46	8.00–6.00	6.60	7.00	7.30	
Left	Girls	6.82 ± 0.48	8.00–6.00	6.50	6.85	7.10	*Z* = − 1.92*p* = 0.054
	Boys	7.00 ± 0.47	8.20–6.00	6.60	7.00	7.40	
Clarke’s angle [°]							
Right	Girls	37.48 ± 11.57	69.00–16.00	31.00	37.50	46.00	*Z* = 3.49*p* = 0.001^*^
	Boys	28.92 ± 11.08	47.00–6.00	20.00	30.00	37.00	
Left	Girls	34.98 ± 11.31	58.00–10.00	27.00	35.50	43.00	*Z* = 3.56*p* < 0.001^*^
	Boys	26.32 ± 10.73	47.00–4.00	17.00	26.50	33.00	
Heel angle *γ* [°]							
Right	Girls	16.84 ± 2.10	20.00–13.00	15.00	17.00	19.00	*Z* = − 0.69*p* = 0.484
	Boys	17.18 ± 1.79	21.00–14.00	16.00	17.00	18.00	
Left	Girls	16.90 ± 2.10	22.00–13.00	15.00	16.50	19.00	*Z* = − 0.52*p* = 0.602
	Boys	17.08 ± 1.85	20.00–14.00	16.00	17.00	18.00	
Hallux valgus angle *α* [°]							
Right	Girls	3.82 ± 3.75	12.00–0.00	0.00	4.00	7.00	*Z* = 0.12*p* = 0.904
	Boys	3.72 ± 3.73	12.00–0.00	0.00	3.50	6.00	
Left	Girls	3.52 ± 3.83	13.00–0.00	0.00	2.00	7.00	*Z* = − 0.92*p* = 0.353
	Boys	4.08 ± 3.42	11.00–0.00	0.00	4.00	7.00	
The angle of the varus deformity of the Vth toe *β* [°]							
Right	Girls	13.64 ± 6.35	25.00–0.00	9.00	15.00	19.00	*Z* = 0.00*p* = 0.997
	Boys	13.64 ± 5.63	24.00–0.00	11.00	14.00	17.00	
Left	Girls	14.92 ± 4.94	25.00–0.00	12.00	15.00	20.00	*Z* = 1.13*p* = 0.259
	Boys	13.66 ± 5.10	22.00–0.00	11.00	13.50	17.00	

**Notes.**

x arithmetical mean value SD standard deviation max maximum value min minimum value Q_25_ lower quartile Me median Q_75_ upper quartile *Z* value of the Mann-Whitney *U* test statistic *p* probability value

* *α* = 0.05.

The data in Table 2 indicate that gender was not a factor determining the frequency of deformation of the longitudinal (right foot: *p* = 0.857; left foot: *p* = 0.577) and transverse (right foot: *p* = 0.331; left foot: *p* = 0.669) foot arch, as well as the position of the hallux (right foot: *p* = 0.461; left foot: *p* = 0.695) and the Vth toe of the left foot (*p* = 1.000), but it determined the frequency of varus deformity of the Vth toe of the right foot (*p* = 0.045). Varus of the Vth toe of the right foot was reported more frequently in boys.

**Table 2:** Frequency of foot deformities depending on the gender of the studied children.

Variable		Girls		Boys		Chi-square test
		*n*	%	*n*	%	
The medial longitudinal arch based on the Clarke’s angle reference values for girls: 29–49° and for boys: 20–44° (Lizis, 2000)						
Right foot	Normal foot	35	70.0	34	68.0	*χ*^2^(2) = 0.31*p* = 0.857
	Flat foot	10	20.0	12	24.0	
	High arched foot	5	10.0	4	8.0	
Left foot	Normal foot	31	62.0	33	66.0	*χ*^2^(2) = 1.10*p* = 0.577
	Flat foot	13	26.0	14	28.0	
	High arched foot	6	12.0	3	6.0	
Transverse arch based on the heel angle reference values: 15–18° (Lizis, 2000)						
Right foot	Normal foot	28	56.0	35	70.0	*χ*^2^(2) = 2.21*p* = 0.331
	Flat foot	15	30.0	11	22.0	
	High arched foot	7	14.0	4	8.0	
Left foot	Normal foot	30	60.0	34	68.0	*χ*^2^(2) = 0.80*p* = 0.669
	Flat foot	14	28.0	12	24.0	
	High arched foot	6	12.0	4	8.0	
Setting of the hallux based on the hallux valgus angle reference values: 0–9° (Lizis, 2000)						
Right foot	Normal setting	47	94.0	45	90.0	*χ*^2^(1) = 0.54*p* = 0.461
	Hallux valgus	3	6.0	5	5.0	
Left foot	Normal setting	46	92.0	47	94.0	*χ*^2^(1) = 0.15*p* = 0.695
	Hallux valgus	4	8.0	3	6.0	
Setting of the Vth toe based on the angle of the varus deformity of the Vth toe reference values: 0–9° (Lizis, 2000)						
Right foot	Normal setting	14	28.0	6	12.0	*χ*^2^(1) = 4.00*p* = 0.045^*^
	The Vth toe varus deformity	36	72.0	44	88.0	
Left foot	Normal setting	6	12.0	6	12.0	*χ*^2^(1) = 0.00*p* = 1.000
	The Vth toe varus deformity	44	88.0	44	88.0	

**Notes.**

*n* number of subjects % percent of subjects *χ*^2^ value of the Chi-square test statistic *p* probability value

* *α* = 0.05.

There were no statistically significant inter-gender differences in terms of the amount of the functional length and width excess noted for right and left foot (Table 3).

**Table 3:** Comparison of the functional excess of footwear of the studied children.

Foot	Gender	$\bar {x}$ ±SD	Max–min	Q_25_	Me	Q_75_	Mann–Whitney *U* test
Functional length excess [mm]							
Right	Girls	11.42 ± 2.40	16.00–6.00	10.00	12.00	13.00	*Z* = − 1.05*p* = 0.289
	Boys	12.00 ± 2.36	16.00–7.00	10.00	12.00	14.00	
Left	Girls	11.43 ± 2.39	16.00–6.00	10.00	13.00	12.00	*Z* = − 1.06*p* = 0.288
	Boys	12.00 ± 2.36	16.00–7.00	10.00	14.00	12.00	
Functional width excess [mm]							
Right	Girls	2.26 ± 1.61	4.50–(−1.00)	2.00	2.20	3.50	*Z* = 0.38*p* = 0.702
	Boys	2.23 ± 1.30	4.00–(−2.00)	2.00	2.45	3.00	
Left	Girls	2.28 ± 1.57	4.50–(−1.50)	2.00	2.05	3.50	*Z* = 0.40*p* = 0.684
	Boys	2.24 ± 1.27	4.00–(−2.00)	2.00	2.50	3.00	

**Notes.**

*x* arithmetical mean value SD standard deviation max maximum value min minimum value Q_25_ lower quartile Me median Q_75_ upper quartile *Z* value of the Mann-Whitney *U* test statistic *p* probability value

Data in Table 4 show that in terms of length, 62.0% of girls and 60.0% of boys wore properly fitted shoes for the right and left feet. Both in the case of girls and boys, the frequency of using shoes that were too long was higher than the frequency of using shoes that were too short. There were no statistically significant frequencies between wearing too long, too short and properly fitted shoes and gender (right foot: *p* = 0,868; left foot: *p* = 0,868).

**Table 4:** Fit of footwear depending on the gender of the studied children.

**Fit of footwear**	Girls		Boys		Chi-square test
	*n*	%	*n*	%	
Matching the length of the footwear to the right foot					
Too long	15	30.0	17	34.0	*χ*^2^(2) = 0.28*p* = 0.868
Appropriate	31	62.0	30	60.0	
Too short	4	8.0	3	6.0	
Matching the length of the footwear to the left foot					
Too long	15	30.0	17	34.0	*χ*^2^(2) = 0.28*p* = 0.868
Appropriate	31	62.0	30	60.0	
Too short	4	8.0	3	6.0	
Matching the width of the footwear to the right foot					
Too wide	16	32.0	12	24.0	*χ*^2^(2) = 2.19*p* = 0.334
Appropriate	27	54.0	34	68.0	
Too narrow	7	14.0	4	8.0	
Matching the width of the footwear to the left foot					
Too wide	16	32.0	11	22.0	*χ*^2^(2) = 2.92*p* = 0.231
Appropriate	28	56.0	36	72.0	
Too narrow	6	12.0	3	6.0	

**Notes.**

*n* number of subjects % percent of subjects *χ*^2^ value of the Chi-square test statistic *p* probability value

In terms of width, 54.0% of girls and 68.0% of boys wore properly fitted shoes for the right foot, while for the left one: 56.0% of girls and 72.0% of boys. For both girls and boys the frequency of using shoes that are too wide was higher in relation to the frequency of using shoes that are too narrow. There were no statistically significant frequencies between wearing too long, too short and properly selected shoes and gender (right foot: *p* = 0.334; left foot: *p* = 0.231).

Multiple regression models with two variables (functional excess of shoe length and width) explaining the variance of the Clarke’s angle were statistically significant for both girls (right foot: *F* = 10.79; *p* < 0.001 and left foot: *F* = 5.20; *p* = 0.009), and boys (right foot: *F* = 36.17; *p* < 0.001 and left foot: *F* = 35.01; *p* < 0.001). The values of the R^2^ determination coefficients inform that in girls these variables explained a total of 31% of the dependent variable (right foot) and 18% of the dependent variable’s variance (left foot). On the other hand, in boys, the values of R^2^ coefficients were higher and amounted to 61% and 60% of the variance of the dependent variable, respectively. Simple regression in girls showed a statistically significant, negative effect of the functional excess of the shoe length on the values of the Clarke’s angle of the right (r_*p*_=−0.71; *p* < 0.001) and left foot (r_*p*_=−0.32; *p* = 0.028). The slope of the regression line for these variables was: *b* =  − 2.68 (right foot) and *b* =  − 1.54 (left foot) which means that if the shoe length increases by one unit, the Clarke’s angle will decrease by an average of 2.68° (right foot) and 1.54° (left foot). Moreover, a statistically significant negative influence of the functional excess of the shoe width on the values of the Clarke’s angle of the right foot (r_*p*_=−0.24; *p* = 0.012) was noted in girls. The value of the coefficient *b* =  − 0.11 indicates that if the shoe width increases by a unit, the Clarke’s angle will decrease by an average of 0.11°. Also in boys, the simple regression showed a statistically significant, negative effect of the functional excess of the shoe length on the Clarke angle values of the right (r_*p*_=−0.53; *p* < 0.001) and left foot (r_*p*_=−0.75; *p* < 0.001), and the values of the b coefficients indicate that if the shoe length increases by one unit, the Clarke’s angle will decrease by an average of 3.35° (right foot) and at 3.32° (left foot). Moreover, a statistically significant negative influence of the functional excess of the shoe width on the values of the Clarke’s angle of the left foot (r_*p*_=−0.30; *p* = 0.034) was found in boys, and the value of the *b* coefficient informs that if the shoe width increases by one unit, the Clarke’s angle will decrease by an average of 1.71° (Table 5).

**Table 5:** Regression models in which the dependent variables are the foot structure indices.

Excess of footwear [mm]		*R*	*R* ^2^	${R}_{\mathrm{adj}}^{2}$	*F*	*p*	*b*	*r*_*p*_	*p*
Clarke’s angle [°] of the right foot									
Girls	Length	0.56	0.31	0.29	10.79	<0.001^*^	−2.68	−0.71	<0.001^*^
	Width						−0.11	−0.24	0.012^*^
Boys	Length	0.78	0.61	0.59	36.17	<0.001^*^	−3.35	−0.53	<0.001^*^
	Width						−2.06	−0.02	0.906
Clarke’s angle [°] of the left foot									
Girls	Length	0.43	0.18	0.15	5.20	0.009^*^	−1.54	−0.32	0.028^*^
	Width						−1.27	−0.18	0.224
Boys	Length	0.77	0.60	0.58	35.01	<0.001^*^	−3.32	−0.75	<0.001^*^
	Width						−1.71	−0.30	0.034^*^
Heel angle (*γ*) [°] of the right foot									
Girls	Length	0.68	0.50	0.44	19.95	<0.001^*^	−0.38	−0.48	<0.001^*^
	Width						0.92	0.66	<0.001^*^
Boys	Length	0.78	0.61	0.59	36.25	<0.001^*^	−0.18	−0.35	0.013^*^
	Width						1.06	0.77	<0.001^*^
Heel angle (*γ*) [°] of the left foot									
Girls	Length	0.71	0.51	0.49	24.32	<0.001^*^	−0.34	−0.45	0.001^*^
	Width						1.02	0.71	<0.001^*^
Boys	Length	0.70	0.49	0.47	22.74	<0.001^*^	−0.03	−0.05	0.710
	Width						1.02	0.70	<0.001^*^
Hallux valgus angle (*α*) [°] of the right foot									
Girls	Length	0.05	0.00	−0.04	0.07	0.937	−0.05	−0.03	0.822
	Width						−0.06	−0.03	0.858
Boys	Length	0.21	0.04	0.00	1.04	0.361	−0.28	−0.17	0.216
	Width						0.34	0.12	0.401
Hallux valgus angle (*α*) [°] of the left foot									
Girls	Length	0.10	0.01	−0.03	0.23	0.788	−0.14	−0.09	0.560
	Width						0.21	0.08	0.577
Boys	Length	0.16	0.02	−0.02	0.58	0.559	−0.22	−0.15	0.284
	Width						0.00	0.00	0.993
The angle of the varus deformity of the Vth toe (*β*) [°] of the right foot									
Girls	Length	0.08	0.01	−0.03	0.17	0.847	−0.23	−0.08	0.570
	Width						0.17	0.04	0.777
Boys	Length	0.18	0.03	−0.01	0.81	0.448	0.41	0.17	0.236
	Width						−0.35	−0.08	0.573
The angle of the varus deformity of the Vth toe (*β*) [°] of the left foot									
Girls	Length	0.20	0.04	−0.00	0.98	0.381	−0.24	−0.11	0.449
	Width						0.67	0.20	0.174
Boys	Length	0.10	0.01	−0.03	0.23	0.797	0.20	0.09	0.526
	Width						0.10	0.02	0.867

**Notes.**

*R* coefficient of multiple correlation *R*^2^ coefficient of determination ${R}_{\mathrm{adj}}^{2}$ adjusted *R*^2^ *F* value of the Fisher-Snedecor test statistic *b* coefficient of slope of the regression line *r*_*p*_ partial correlation *p* probability value

* *α* = 0.05.

The influence of predictive variables on the values of the heel angle (*γ*) was statistically significant for both girls (right foot: *F* = 19.95; *p* < 0.001 and left foot: *F* = 24.32; *p* < 0.001) and boys (right foot: *F* = 36.25; *p* < 0.001 and left foot: *F* = 22.74; *p* < 0.001). The values of the R^2^ coefficients indicate that in girls, these variables explained a total of 50% of the variance of the dependent variable (right foot) and 51% of the variance of the dependent variable (stopa lewa), while in boys they accounted for 61% and 49% of the variance of the dependent variable, respectively. Simple regression in girls showed a statistically significant, negative effect of the functional excess of shoe length on the values of the heel angle (*γ*) of the right (r_*p*_=−0.48; *p* < 0.001) and left (r_*p*_=−0.45; *p* = 0.001) foot, and the values of the b coefficients indicate that if the shoe length increases by one unit, the heel angle (*γ*) will decrease by an average of 0.38° (right foot) and 0.34° (left foot). In girls, a statistically significant, positive effect of the functional excess of footwear width on the values of the heel angle (*γ*) of the right (r_*p*_ = 0.66; *p* < 0.001) and left (r_*p*_ = 0.71; *p* < 0.001) foot was also noted. The *b*-factor values indicate that if the shoe width increases by one unit, the heel angle (*γ*) will increase by an average of 0.92° (right foot) and 1.02° (left foot). Simple regression in boys showed a statistically significant, negative effect of the functional excess of shoe length on the values of the Clarke’s angle of the right foot (r_*p*_=−0.35; *p* = 0.013), and the values of the b coefficients indicate that if the shoe length increases by one unit, the Clarke angle will decrease by an average of 0.18°. In addition, in boys, a statistically significant, positive effect of the functional excess of the footwear width on the values of the heel angle (*γ*) of the right (r_*p*_ = 0.77; *p* < 0.001) and left (r_*p*_ = 0.70; *p* < 0.001) foot was found, and the values of the *b* coefficients indicate that if the shoe width increases by one unit, the heel angle (*γ*) will increase by an average of 1.06° (right foot) and by 1.02° in the case of left foot (Table 5).

Multiple regression models with two variables explaining the variance of the hallux valgus angle (*α*) as well as the variance of the angle of the varus deformity of the Vth toe (*β*) were statistically insignificant (Table 5).

## Discussion

In our material, gender differentiated the values of Clarke’s angle, which were lower in the case of boys. This suggests that the development of the medial longitudinal arch may be slower in them than in girls. Similar conclusions were drawn by Prętkiewicz-Abacjew & Opanowska (2013) based on studies of children from randomly selected kindergartens in northern Poland. In turn, Delgado-Abellán et al. (2014) found no gender differences in the height of the longitudinal arch of the foot in 6-year-old Spanish children. We found that the values of the remaining foot construction indices did not differentiate girls and boys. Comparative data on the feet of 6-year-old girls and boys appear in the literature in a fragmentary way, hence it is difficult to relate our results to the reports of other authors. Few studies available include Delgado-Abellán et al. (2014) and Bosch, Gerss & Rosenbaum (2010) works which also found no gender differences in foot length in 6-year-olds.

We found that the feet of approximately 60% of girls and boys had correct longitudinal and transverse arches, and over 90% of the respondents had the correct position of the hallux. It is worth emphasizing that gender was not a factor determining the frequency of deformation of the longitudinal and transverse arch of the foot, as well as the position of the hallux. The feet of 70% of the examined children were characterized by a varus position of the Vth toe, and the gender determined the frequency of this abnormality in the right foot. Boys were characterized by more frequent varus position of the Vth toe than girls. In preschool children, regardless of gender, Knapik & Mazur (2003) observed a tendency to increased varus angle of the Vth toe to a greater degree than that of valgus angle of the hallux. The authors presumed that the cause of these deformations may be wearing inadequately fitted, too narrow footwear. Our study, on the other hand, ruled out the influence of shoe length and width on the position of the hallux and Vth toe in the examined children, which will be analyzed in detail further in the Discussion.

Our study indicates that the functional excess of the length and width of indoor footwear did not differentiate the examined girls and boys. There are no reports in the literature on this type of intergender comparisons, so these results are difficult to relate to the results of other authors.

In our study, about 60% of children wore correctly fitted footwear in terms of length and width. Both in the case of girls and boys, the incidence of wearing too long and too wide shoes was higher in relation to the frequency of wearing too short and too narrow shoes. Perhaps the high growth rate of the feet length in this period is the reason why parents decide to buy shoes longer than the size of children’s feet, so that they “last longer”. However, such a procedure is unjustified, especially taking into account the fact that the analyzed type of footwear requires frequent replacement due to relatively quick wear. The obtained data may contribute to further scientific research, which may include asking parents about the cause of this trend. González Elena & Córdoba-Fernández (2019) obtained different results compared to ours, as 72.5% of the children from Seville (Spain) wore shoes that were too short, and 66.7% of children used shoes that were too narrow. However, these data are difficult to relate to our results, due to the fact that the authors analyzed them for a wide range of ages from 3 to 12 years, without a detailed analysis by year. Delgado-Abellán et al. (2014), came to interesting conclusions as a result of comparison between the real size of the shoe and the size estimated from the foot length in children aged 6–12. The authors observed that there were no significant differences in boys between the real size and the estimated size, however, there were some differences in girls. It seems that the girls wore shoes that were too small for the length of their feet. The obtained results led the authors to the conclusion that most footwear designs do not take into account the need for matching different widths for the same length. Research by Klein et al. (2009) on the adjustment to the length of indoor footwear in preschoolers aged 3 to 6.5 from Austria indicated a small percentage of correctly fitted indoor footwear. In many cases, the footwear was too short and not exchanged throughout the school year. The tendency of children to wear too short shoes in the early stages of ontogenesis was highlighted by Zhang & Wang (2017), as well as Yurt, Sener & Yakut (2014) as a result of research by Turkish preschool-aged children. The authors suggest that parents should ensure that they purchase properly selected children’s footwear and check its fit every two months.

Our research is one of few papers in the field of footwear impact on foot formation in girls and boys in early childhood. The study of the impact of functional excess of indoor footwear on the foot shape showed that in both sexes, the length and width of shoes affect longitudinal and transverse arches. The longer and wider the shoes, the lower the length of the arch. Longer shoes are accompanied by a greater transverse arch, and wider ones—a lower transverse arch of the foot. This suggests that due to the lowering of the longitudinal and transverse arch of the foot, children require footwear which is longer and wider and therefore their shoes need to be bigger in size. On the other hand, the size of the functional excess of the length and width of indoor footwear did not affect the position of the hallux and Vth toe of the examined children. Our results contradict those of Klein et al. (2009) who observed an increased risk of hallux valgus in preschool children wearing too short shoes.

### Strengths and limitations of the study

The present study is well anchored in our previously published investigations on impact of specific type of footwear regularly worn on foot morphology in children and adults. Is the multi-faceted assessment of the effect of wearing shoes. The key causative factors of health-hazardous deformities to the children’s feet, including instrumental in the occurrence of potential locator problems were established which offers a distinct guidelines for public health policy. The study population was homogeneous of, *i.e.*, representative of a substantial proportion of the population of 6-year-olds from children in the Tuchow district and typical footwear worn on the regular basis at the kindergarten (indoor footwear), ensures credibility of the results. The actual allocation of subjects to the study, pursued in line with the adopted inclusion criteria, on the one hand, allowed to ensure homogeneity within a group, fully corresponding to pertinent characteristics of 6-year-olds from Tuchow population, while on the other, resulted in reducing the number of potential recruits, which might well be regarded as a study limitation. The present results would gain even more credibility, when compared with the ones based on a much larger study sample. Taking into account the general importance and the sheer scale of the issue under study, any subsequent reports would contribute significantly to its distinction, while at the same time giving it the importance it deserves in scientific research.

## Conclusions

1. The boys’ feet had a lower longitudinal arch. Gender was not the factor determining the frequency of deformation of the longitudinal and transverse foot arch, as well as the position of the hallux and Vth toe of the left foot, but it determined the frequency of varus deformities of the Vth toe of the right foot. Varus of the Vth toe of the right foot was reported more frequently in boys.
2. The functional excess of the length and width of the footwear did not differentiate girls and boys.
3. About 60% of children wore correctly fitted shoes in terms of length and width. Both in the case of girls and boys, the frequency of using too long and too wide shoes was higher in relation to the frequency of using too short and too narrow shoes. The gender did not determine the length and width of the shoes.
4. The length and width of the shoes affected the length and width of the footwear both in girls and boys. The longer and wider the shoes, the lower the length of the arch. Longer shoes are accompanied by a greater transverse arch, and wider ones with a lower transverse arch of the foot. This suggests that due to the lowering of the longitudinal and transverse arch of the foot, children require footwear which is longer and wider and therefore their shoes need to be bigger in size. The size of the functional excess of the length and width of indoor footwear did not affect the position of the hallux and Vth toe.

##  Supplemental Information

10.7717/peerj.13403/supp-1 Supplemental Information 1 Codebook

10.7717/peerj.13403/supp-2 Supplemental Information 2 Raw data for Tables 1–5

## Additional Information and Declarations

### Data Availability

The following information was supplied regarding data availability: The raw measurements are available in the Supplementary File.
