# Supplementary material for: Impact of length and width of footwear on foot structure of preschool-aged children
Source: PeerJ. 2022 May 3;10:e13403. doi: 10.7717/peerj.13403 (PMC9074857; doi:10.7717/peerj.13403)
Supplement: Supplemental Information 1 [file peerj-10-13403-s001.docx]

Sex:

1 – girl

2 – boy

Length excess:

8-12 – appropriate

< 8 – too short

> 12 – too long

Width excess:

1-3 – appropriate

< 1 – too narrow

> 3 – too wide
